# Exercise self-efficacy remains unaltered during military service

**DOI:** 10.3389/fpsyg.2024.1307979

**Published:** 2024-01-29

**Authors:** Tiia Kekäläinen, Antti-Tuomas Pulkka, Heikki Kyröläinen, Tommi Ojanen, Joonas Helén, Kai Pihlainen, Risto Heikkinen, Jani P. Vaara

**Affiliations:** ^1^Faculty of Sport and Health Sciences, University of Jyväskylä, Jyväskylä, Finland; ^2^Department of Leadership and Military Pedagogy, National Defence University, Helsinki, Finland; ^3^Human Performance Division, Finnish Defence Research Agency, Tuusula, Finland; ^4^Training Division, Defence Command, Finnish Defence Forces, Helsinki, Finland; ^5^Statistical Analysis Services, Analyysitoimisto Statisti Oy, Jyväskylä, Finland

**Keywords:** physical activity, exercise, self-efficacy, young adults, military, intervention

## Abstract

**Background:**

Exercise self-efficacy is a crucial aspect of adopting and maintaining a physically active lifestyle. Regular physical activity may enhance exercise self-efficacy. This study aimed to investigate the baseline associations of physical fitness, physical activity, and body composition with exercise self-efficacy and the effects of military service on exercise self-efficacy. Methods: The sample consisted of healthy young Finnish conscripts (*n* = 243) undergoing military service. The participants were divided into two groups: an intervention group undergoing a high-intensity functional training program (*n* = 113) and a control group undergoing traditional physical training within their military service (*n* = 130). Exercise self-efficacy (adoption and barrier) and aerobic and muscular fitness were measured thrice (baseline, month 3, and month 5). Self-reported leisure-time physical activity and measured fat percentage were collected at baseline.

**Results:**

Adoption and barrier exercise self-efficacy correlated positively with aerobic and muscular fitness and leisure time physical activity (*r* = 0.33–0.59, *p* < 0.001), and barrier self-efficacy negatively with fat percentage (*r* = −0.15, *p* < 0.05) at baseline. No changes in adoption (time *p* = 0.912) and barrier self-efficacy (time *p* = 0.441) occurred during the military service. There were no differences between groups in these changes (group × time interaction *p* = 0.643 for adoption self-efficacy and *p* = 0.872 for barrier self-efficacy). Change in muscular fitness correlated positively with change in barrier self-efficacy in the high-intensity functional training group (*r* = 0.35, *p* < 0.05). Conclusions: Exercise self-efficacy is positively associated with physical fitness and physical activity among young males. However, military service, whether it involves high-intensity functional physical training or more diverse traditional physical training, does not improve exercise self-efficacy.

## 1 Introduction

The decline in physical activity from adolescence to adulthood is a risk factor for many later health problems (Corder et al., 2019). The transition from higher secondary education (i.e., high school or vocational school) to university-level education or employment is a critical point for physical activity decline as school-based physical activity promotion is left behind (Winpenny et al., 2020). Therefore, physical activity promotion interventions should target this transition phase to support the maintenance of a physically active lifestyle and physical fitness. There is a growing public health concern about promoting physical activity, particularly among young males, as their aerobic capacity and muscular fitness have declined and the prevalence of obesity has increased over the past few decades (Santtila et al., 2018).

Physical fitness is a key attribute for soldiers to successfully complete their duties in a given operation or mission (Friedl et al., 2015). Military tasks commonly require an adequate level of aerobic and muscular fitness (Vaara et al., 2022). Therefore, it is essential for soldiers to engage in physical activity and training throughout their military career to maintain and develop their physical fitness characteristics. At the beginning of the military service, one of the main objectives is to educate soldiers about physical training, including its planning, implementation, and monitoring. Although previous studies have reported changes in physical fitness levels during military service (Pihlainen et al., 2020), less is known about how physical education within the military service contributes to psychological factors related to exercise, such as self-efficacy.

Self-efficacy reflects a person’s beliefs in their capabilities to succeed in a particular situation or task (Bandura, 1994). These judgments of capabilities play an important role in both the choice of activities and the effort put into the activities. Self-efficacy is a task-specific orientation (Bandura, 1994), and different types of self-efficacy are needed in different stages of behavior change, such as in the adoption of exercise behavior and in confronting different barriers to maintaining it (Schwarzer and Renner, 2000; Schwarzer, 2008).

A bidirectional relationship exists between exercise behavior and exercise self-efficacy: exercise self-efficacy can be both a determinant and an outcome of exercise behavior (McAuley and Blissmer, 2000). The change in self-efficacy predicts the change in actual behavior (Sheeran et al., 2016) and physical activity interventions are an effective way to improve exercise self-efficacy (Higgins et al., 2014). Even though interventions including psychological techniques (e.g., planning and feedback) may be more effective in improving exercise self-efficacy (Ashford et al., 2010; Williams and French, 2011), interventions focusing only on physical activity may also be advantageous, but are less studied (Higgins et al., 2014). The positive effect of physical activity on exercise self-efficacy may be due to many reasons, such as obtained mastery experiences, enjoyment of physical activity, or gained physical benefits (McAuley and Blissmer, 2000).

Men are often underrepresented in health behavior research (Robertson et al., 2008; Ryan et al., 2019), and health behavior interventions are biased toward participants who already have at least some motivation to change their behavior. Also studies assessing changes in exercise self-efficacy are mainly conducted among middle-aged adults and female samples (Higgins et al., 2014; Ghayour Baghbani et al., 2023). In Finland, military service is compulsory for male citizens and all men take part in a call-up in the year they turn 18 years (The Finnish Defense Forces, 2020). In general, military service is carried out right after higher secondary education at the age of 19–20, and approximately 70% of the male population undergoes military service in Finland. Military service includes strenuous physical training and has many beneficial effects on functional capacity and cardiovascular risk factors, such as improvements in endurance and muscle fitness, a decrease in fat mass, and an increase in lean body mass (Mikkola et al., 2009; Cederberg et al., 2011). At the same time, it offers a unique opportunity to reach most of the age cohort of young males and expose them to physical activity intervention within the military service.

The purpose of the present study was to investigate the correlates of exercise self-efficacy at the beginning of military service and the effects of military service on exercise self-efficacy. This is a secondary analysis of a non-randomized controlled trial. The main aim of the trial was to compare the effectiveness of concurrent strength and endurance training with an emphasis on high-intensity functional training to traditional exercise training during military service (Helén et al., 2023). To our knowledge, this is the first study to investigate the effects of physical training on exercise self-efficacy in military service.

## 2 Methods

### 2.1 Participants

Participants were drawn from a sample of Finnish male conscripts starting military service in June 2019. In total, 243 voluntary males participated in the study. They were 19 ± 1.0 years old and did not have previous military experience.

**Figure 1:**
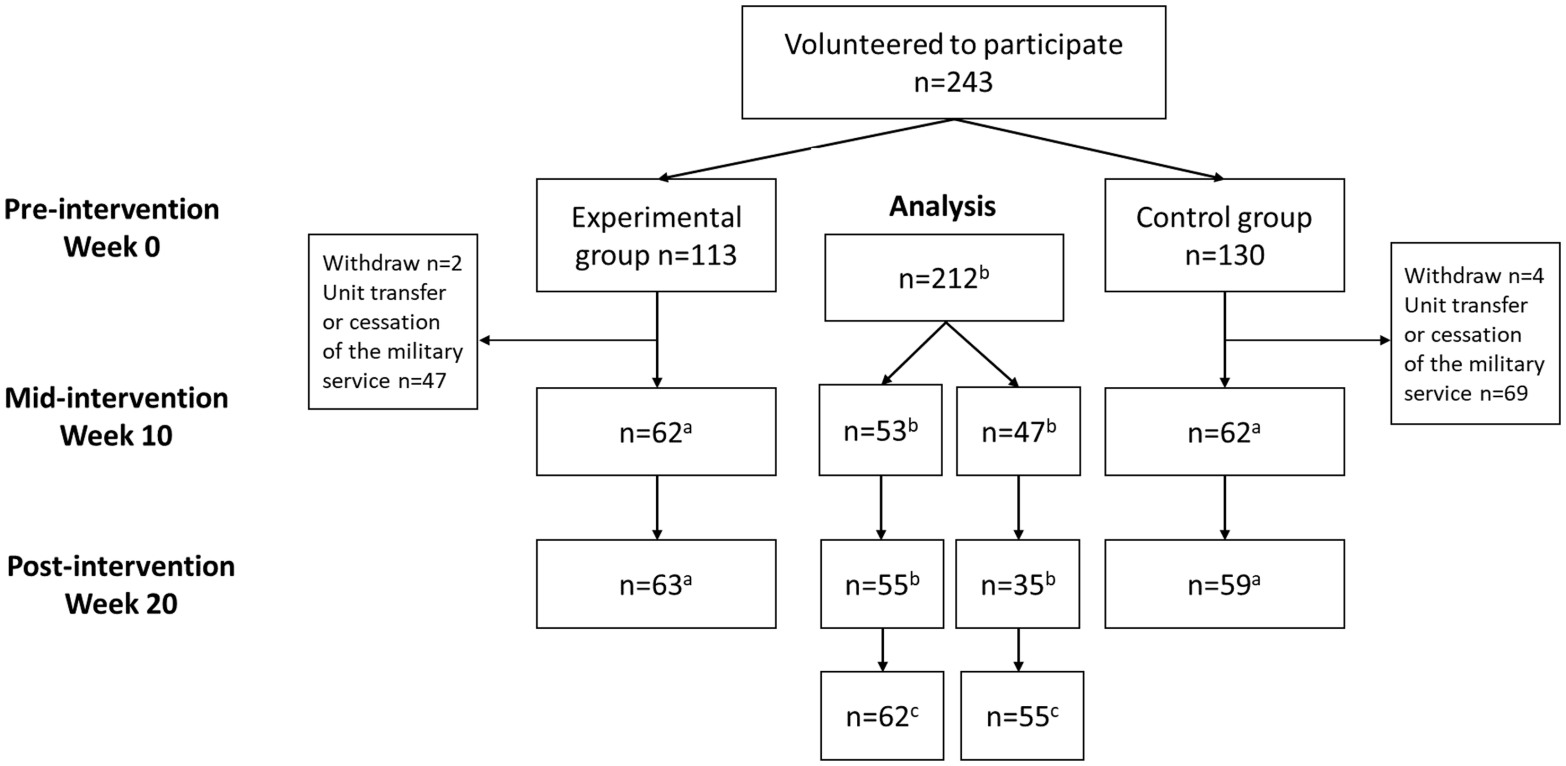
The flow of the participants. ^a^Some participants were not able to participate in assessments due to other military duties, ^b^Self-efficacy information available, ^c^Included in the analysis of intervention effects.

The flow of the participants is presented in Figure 1. Two companies were selected to an experimental intervention group undergoing a high-intensity functional training program (*n* = 113), and a control group undergoing traditional physical training within their military service (*n* = 130). Six participants withdrew their participation from the study, and 116 dropped out due to transfer to another unit or cessation of military service. Pre-intervention measurements were conducted a week before starting the intervention, mid-intervention measurements at week 10, and post-intervention measurements a week after the intervention at week 20.

The study was approved by the Ethical Committee of the Central Finland Health Care District (HUS-1557-2018-8) and the Finnish Defense Forces (AP10027). All participants provided written informed consent to participate.

### 2.2 Intervention

The intervention is described in more detail in Helén et al. (2023). The experimental group participated in supervised strength and endurance training with an emphasis on high-intensity functional training while the control group followed the current physical training guidelines of the Finnish Defense Forces for 19 weeks. Some training objectives were necessary for both groups (e.g., swimming, running, and orienteering) and thus, 16 h of a total of 46 training hours in the experimental group were identical to the control group. All training sessions were supervised by instructors.

The training sessions in the experimental group included a standardized warm-up (a short low-intensity aerobic exercise, dynamic stretching, and core stability exercises), one or two strength exercises, and a high-intensity functional training part. Strength exercises included various exercises (e.g., squats, deadlifts, push-ups, shoulder presses, sit-ups), and the number of sets and repetitions as well as the length of rest period varied between training sessions. Similarly, the intensity, duration, and exercises in a high-intensity functional training part varied. Most of the workouts included performing a prescribed number of sets and repetitions as fast as possible or as many rounds as possible in a given time frame. Body weight, adjustable sandbags from 10 to 60 kg (Brute Force Training LLC, Arvada, CO, USA), and kettlebells from 16 to 32 kg were utilized in the training sessions. The overall volume of both strength exercises and high-intensity interval training was increased progressively throughout the training period. The total amount of training during the intervention was 33 training sessions and 46 h, and the mean participation was 88 ± 9%.

Training in the control group was planned in accordance with the physical training guidelines of the Finnish Defense Forces. The training sessions focused on traditional military exercises, such as running, ball games, and calisthenics. The total amount of training during the intervention period was 35 training sessions and 42 h, and the mean participation was 91 ± 8%.

In addition to physical training, both groups participated in similar normal military training including live fire exercises, weapons and equipment training, combat training, theoretical education, and cross-country marches.

### 2.3 Measurements

The questionnaire (including demographics, exercise self-efficacy and leisure time physical activity) was assessed a day before when baseline physical fitness measurement took place. Body composition was assessed after an overnight fast in the morning before physical fitness tests. Seated medicine ball throw, isometric bench press, and isometric leg press were assessed in the morning and other muscular fitness tests (standing long jump, sit-ups, push-ups) were assessed in the afternoon. Aerobic fitness test was assessed in the afternoon on an alternative day than muscular fitness tests.

Exercise self-efficacy was measured by 10 items with a question: “How certain are you that you could overcome the following barriers? I can manage to carry out my exercise intentions…” Five items evaluated self-efficacy related to the adoption of exercise behavior (e.g., “even if I have to make a detailed plan to exercise”) (Schwarzer and Renner, 2000) and five items barrier self-efficacy (e.g., “even when I am busy”) (Schwarzer and Renner, 2009). The replies were given on a scale from 1 = *very certain I cannot* to 4 = *very certain I can*, and aggregated. The Cronbach’s alphas were 0.84 for adoption self-efficacy and 0.88 for barrier self-efficacy.

Leisure time physical activity was measured at baseline with a question assessing the level of leisure time physical activity during the last 3 months. Participants were asked to consider physical activity that lasts at least 20 min per time. The scale was 1 = less than once a week; 2 = no vigorous activities, but light or moderate physical activity at least once a week, 3 = vigorous activity once a week; 4 = vigorous activity twice a week; 5 = vigorous activity 3 times a week; and 6 = vigorous activity at least 4 times a week (Fogelholm et al., 2006; Vaara et al., 2014).

Physical fitness: Aerobic fitness was assessed by the 12-min Cooper test that evaluates the maximal aerobic capacity (Cooper, 1968). The aim is to run as far as possible within 12 min and the result is presented in meters. Muscular fitness was assessed by six tests: sit-ups, push-ups, standing long jump, seated medicine ball throw, isometric bench press, and isometric leg press. Detailed information on test protocols is published earlier (Vaara et al., 2020). Maximum repetitions per minute of sit-ups and push-ups were used to measure the muscular endurance of the trunk and upper extremities. Standing long jump and medicine ball throw were used to assess explosive force production of the lower and upper body. After several practice jumps/throws, the result of the best jump/throw attempt was expressed in centimeters. Isometric bench press and isometric leg press were used to measure the maximal isometric force of the upper and lower extremities. After one practice trial, the best result of two trials was selected. The same measurements for physical fitness were repeated at pre-intervention, mid-intervention, and post-intervention.

Body fat percentage was measured with a bioelectrical impedance analyzer (InBody 720/770; Biospace, Seoul, Korea) in the morning after overnight fasting (at least 7 h).

### 2.4 Statistical analysis

Statistical analyses were performed with R Statistical Software (v. 4.2.3.) (R Core Team, 2023). Means, standard deviations, and Pearson bivariate correlations were used for descriptive purposes. Pearson bivariate correlations were also used to investigate relationships between changes in exercise self-efficacy and changes in physical fitness during military service. Changes were calculated by subtracting the pre-intervention value from the post-intervention value.

The effects of military service on exercise self-efficacy were analyzed by linear mixed models. The mixed modeling approach takes into account that the repeated measures within a participant are correlated and uses full information also from an incomplete number of measurements. Participants that had at least two of three measurements were included in the models. The interaction effect of time and training group was assessed in models including time, group, and the interaction term time × group. Given the all-male sample and virtual lack of variance in age, gender, and age were excluded from covariates. The standardized effect sizes for changes within groups were calculated with Cohen’s d formula (Cohen, 1992). The effect sizes of 0.2–0.5 are considered small, 0.5–0.8 are considered medium, and > 0.8 are considered large.

We did post-hoc power calculations for mean group differences in changes of adoption and barrier self-efficacy. We used G*Power (v. 3.1) as a tool with sample variances and assumed a minimal substantial clinical difference of 0.5 points. For simplicity, we calculated the powers separately for different time points as the group sizes differ between time points. The analysis indicated a power of ≥0.97 for both time points and both self-efficacy variables.

## 3 Results

Descriptive statistics and bivariate correlations between study variables at baseline are shown in Table 1. Both adoption and barrier self-efficacy had moderate positive correlations (*r* = 0.33–0.59, *p* < 0.001) with physical activity and physical fitness, and mild negative correlations with fat percentage (*r* = −0.12–0.15, *p* < 0.05).

**Table 1:** Study variables at the baseline.

	n	M	SD	Correlations				
				2	3	4	5	6
1. Adoption self-efficacy	212	2.92	0.60	0.65***	0.46***	0.33***	0.42***	−0.12
2. Barrier self-efficacy	211	2.75	0.68		0.59***	0.39***	0.35***	−0.15*
3. Physical activity	208	3.63	1.66			0.53***	0.45***	−0.24***
4. Aerobic fitness (m)	212	2,279	335				0.54***	−0.61***
5. Muscular fitness	230	0.02	0.79					−0.34***
6. Body fat percentage	225	15.8	7.57					

***p < 0.001, *p < 0.05, M, mean; SD, standard deviation.

### 3.1 Effects of military service on exercise self-efficacy

The effects of military service on exercise self-efficacy are shown in Table 2. No changes in adoption and barrier self-efficacy occurred during military service (Table 2).

**Table 2:** The effect of the intervention on exercise self-efficacy, analyzed by linear mixed models (experimental group *n* = 62, control group *n* = 55).

	Pre	Mid	Post	Effect size Pre-Post	Time	Group	Time × Group
	M ± SD	M ± SD	M ± SD	ES ± SE	F (df), p	F (df), p	F(df), p
Adoption self-efficacy							
EG	2.98 ± 0.65	2.95 ± 0.64	3.00 ± 0.62	0.03 ± 0.17	0.09 (2), 0.912	4.69 (1), 0.032	0.44 (2), 0.643
CG	2.76 ± 0.53	2.78 ± 0.67	2.76 ± 0.65	0.00 ± 0.19			
Barrier self-efficacy							
EG	2.85 ± 0.63	2.81 ± 0.68	2.87 ± 0.64	0.03 ± 0.17	0.82 (2), 0.441	6.29 (1), 0.014	0.14 (2), 0.872
CG	2.58 ± 0.68	2.57 ± 0.76	2.58 ± 0.69	0.00 ± 0.19			

EG, Experimental group; CG, Control group; M, mean; SD, standard deviation; ES, effect size; SE, standard error.

When comparing a high-intensity functional training program with traditional military service (Table 2) high-intensity functional training group had higher scores in both adoption and barrier self-efficacy. No significant changes in time or group × time interactions were found.

Pearson bivariate correlations between the changes in self-efficacy and changes in fitness are shown in Table 3 and scatter plots in Supplementary Figures S1–S4 separately for groups. An increase in muscular fitness was associated with an increase in barrier self-efficacy (*r* = 0.35, *p* < 0.05) in the high-intensity functional training group. No other associations were found.

**Table 3:** Pearson bivariate correlations between pre-post changes in self-efficacy and fitness.

Pre-post change in	1.	2.	3.	4.
1. Adoption self-efficacy	-	0.32*	0.19	0.09
2. Barrier self-efficacy	0.52**	-	0.23	0.35*
3. Aerobic fitness	−0.0.04	−0.03	-	0.49***
4. Muscular fitness	0.13	−0.01	0.26*	-

*p < 0.05, **p < 0.01, ***p < 0.001.

The high-intensity functional training group above and the control group below diagonal.

## 4 Discussion

The purpose of the present study was to investigate the correlates of exercise self-efficacy among young males and the effects of military service on exercise self-efficacy. The results indicated that physically active young males with better physical fitness had higher exercise self-efficacy than those who were less physically active and had poorer physical fitness. Military service including either high-intensity functional physical training or more diverse traditional physical training did not affect exercise self-efficacy. However, changes in muscular fitness were positively associated with changes in barrier self-efficacy in the high-intensity physical training group.

Consistent with the literature (McAuley and Blissmer, 2000; Imayama et al., 2013; Medrano-Ureña et al., 2020; Han et al., 2022), this research found that exercise self-efficacy is positively associated with leisure time physical activity, and physical fitness and negatively with body fat percentage. Although our analysis does not provide an opportunity for causal interpretations, these relationships are likely bidirectional: good exercise self-efficacy leads to gains in physical fitness and body composition through participation in leisure time physical activity, and regular participation in leisure time physical activity and good physical fitness maintain good exercise self-efficacy (McAuley and Blissmer, 2000; Imayama et al., 2013; Higgins et al., 2014; Sheeran et al., 2016; Medrano-Ureña et al., 2020). In addition, this study found a positive association between changes in barrier self-efficacy and aerobic fitness. Even though there was no general improvement in self-efficacy during the intervention, participants with higher improvements in muscular fitness were more likely to improve their barrier self-efficacy in the high-intensity functional training group. This is in line with the idea that exercise self-efficacy may reflect changes in physical fitness (Medrano-Ureña et al., 2020). Those with the lowest baseline fitness level and self-efficacy likely improved the most during the high-intensity functional training program because they were further from their maximum fitness potential. It has been shown that combined strength and endurance training improves both muscular and aerobic fitness to a higher extent that either training method alone (Coffey and Hawley, 2017). Similar results have been presented also in military settings (Burley et al., 2020).

Previous studies have found that interventions including physical activity may increase exercise self-efficacy (Ashford et al., 2010; Higgins et al., 2014). It has been suggested that self-efficacy can be improved, for example, by successfully performing the target behavior (mastery experiences) and observing others succeed in the target behavior (vicarious experiences) (Bandura, 1994; Ashford et al., 2010). Regular participation in physical activity for a sufficient duration with similar peers could lead to improvements in exercise self-efficacy. However, this study was unable to demonstrate improvements in exercise self-efficacy, even though conscripts participated in regular supervised physical training for 5 months. This inconsistency with previous studies is likely related to the military service as an intervention environment.

Adoption self-efficacy reflects the ability to start and maintain an exercise routine (Schwarzer and Renner, 2000), and barrier self-efficacy is related to integrating regular physical activity into daily routines and learning to cope with barriers to exercise (Schwarzer, 2008; Higgins et al., 2014). In this study, participants may have viewed physical training as part of military service and not as a routine they could continue at home. These findings support the idea from a previous review that being physically active independently in one’s everyday environment is a more effective way to gain mastery experiences and develop exercise self-efficacy than exercising in heavily structured and supervised sessions (Higgins et al., 2014). Therefore, interventions targeting exercise self-efficacy should focus on participants’ typical environments and integrate voluntary exercise into daily routines (Higgins et al., 2014). This may be especially challenging in military service, which is typically highly structured, and physical education is externally programmed without conscripts` active participation or involvement.

Previous reviews suggest that the most effective behavior change techniques in improving exercise self-efficacy include vicarious experiences, feedback techniques (Ashford et al., 2010), individualized goal setting, (Higgins et al., 2014), and setting exercise plans (Williams and French, 2011). Even though these have not been tested in a military environment, including some behavior change techniques at the end of military service may help shift learned exercise routines into a home environment. Even a single session, including setting exercise goals and making plans for how to continue exercising after military service, may be beneficial. However, it should be kept in mind that military service focuses on many other issues besides developing physical fitness and promoting a physically active lifestyle.

The study is limited by the lack of information on post-military exercise self-efficacy and actual physical activity behavior. It would be important to follow up on whether the exercise routines learned during military service are maintained in a home environment and which characteristics predict successful maintenance. In addition, it was not possible to study the effects of leisure-time physical activity simultaneously with exercise self-efficacy, as post-intervention measures were conducted during the military service. It should also be noted that some participants continued military service after the post-intervention measures for three or 6 months. This may have made reflections about exercise routines outside military service irrelevant or vague given different future time perspectives. A relatively large proportion of participants discontinued the intervention due to a shift to another unit, some discontinued the military service and some were not able to participate in the study assessments due to other military duties. This is a typical situation in military service but may limit the statistical power of the results.

Despite the aforementioned limitations, this study contributes to our understanding of the factors associated with exercise self-efficacy in a relatively representative sample of young Finnish males. The main strength of this study is the relatively random sample of the age cohort of young males with diverse physical activity and physical fitness background. It is likely that the sample also represents those young males that would not typically be interested in participating in interventions.

## 5 Conclusion

The study findings indicate that exercise self-efficacy remains unchanged during military service in conscripts. Moreover, to the best of our knowledge, this is the first study to investigate the impact of physical training on exercise self-efficacy in military service. In conclusion, physically active young men with better physical fitness tend to have higher exercise self-efficacy, and physical training during military service does not appear to affect exercise self-efficacy. Inclusion of setting exercise goals and making plans for how to continue exercising after military service could potentially facilitate the transition of exercise routines to a home environment. Future studies should investigate strategies for promoting exercise self-efficacy during military service, as well as for maintaining the exercise routines in the long term after military service.

## Data availability statement

The datasets presented in this article are not readily available because the Finnish Defense Forces own and manage the data which are available for researchers who meet the criteria for access to confidential data. Requests to access the datasets should be directed to Jani.Vaara@mil.fi.

## Ethics statement

The studies involving humans were approved by the Ethical Committee of the Central Finland Health Care District (HUS-1557-2018-8) and the Finnish Defense Forces (AP10027). The studies were conducted in accordance with the local legislation and institutional requirements. The participants provided their written informed consent to participate in this study.

## Author contributions

TK: Conceptualization, Writing – original draft, Visualization, Methodology. A-TP: Writing – review & editing, Conceptualization. HK: Writing – review & editing, Funding acquisition, Project administration. TO: Writing – review & editing, Conceptualization, Project administration. JH: Writing – review & editing, Conceptualization. KP: Writing – review & editing, Conceptualization. RH: Formal analysis, Writing – review & editing, Data curation, Visualization. JV: Conceptualization, Writing – review & editing, Project administration.
