# Supplementary material for: Exercise self-efficacy remains unaltered during military service
Source: Front Psychol. 2024 Jan 29;15:1307979. doi: 10.3389/fpsyg.2024.1307979 (PMC10859872; doi:10.3389/fpsyg.2024.1307979)
Supplement: Supplementary file 1 [file Data_Sheet_1.PDF]

Supplementary Material

Exercise Self-Efficacy Remains Unaltered During Military Service

Tiia Kekäläinen*, Antti-Tuomas Pulkka, Heikki Kyröläinen, Tommi Ojanen, Joonas Helén, Kai Pihlainen, Risto Heikkinen, Jani P Vaara

*** Correspondence:** Tiia Kekäläinen: tiia.m.kekalainen@jyu.fi

Supplementary Figures

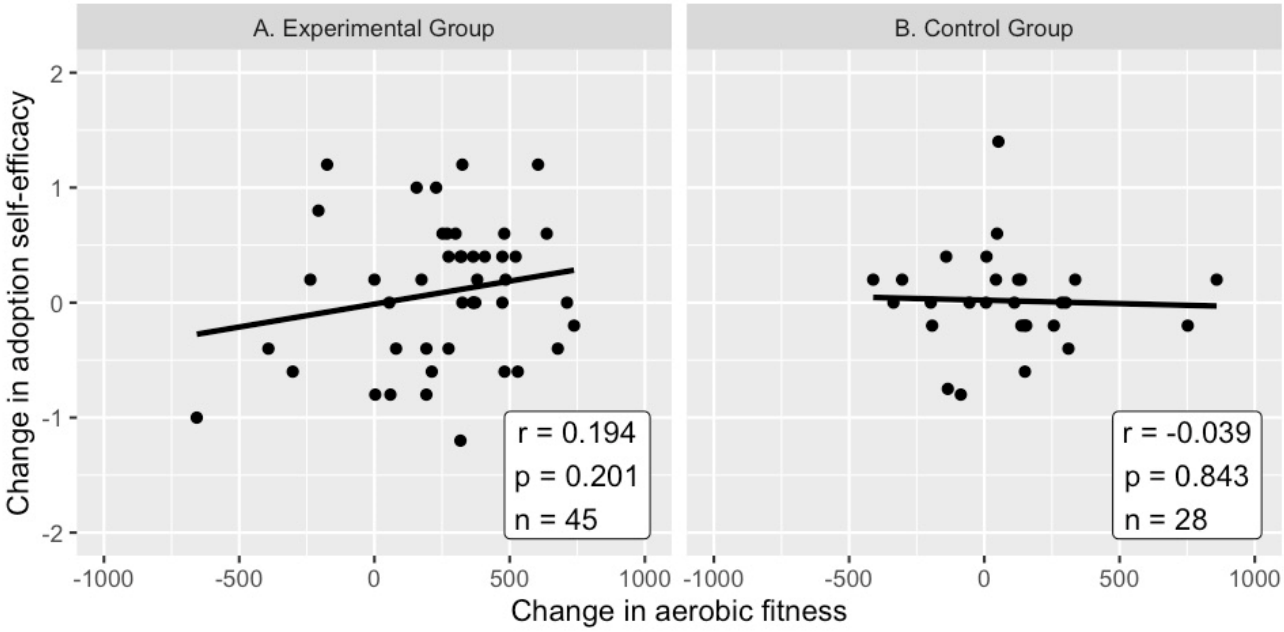

Supplementary Figure 1. A scatter plot for the association between changes in adoption self-efficacy and aerobic fitness.

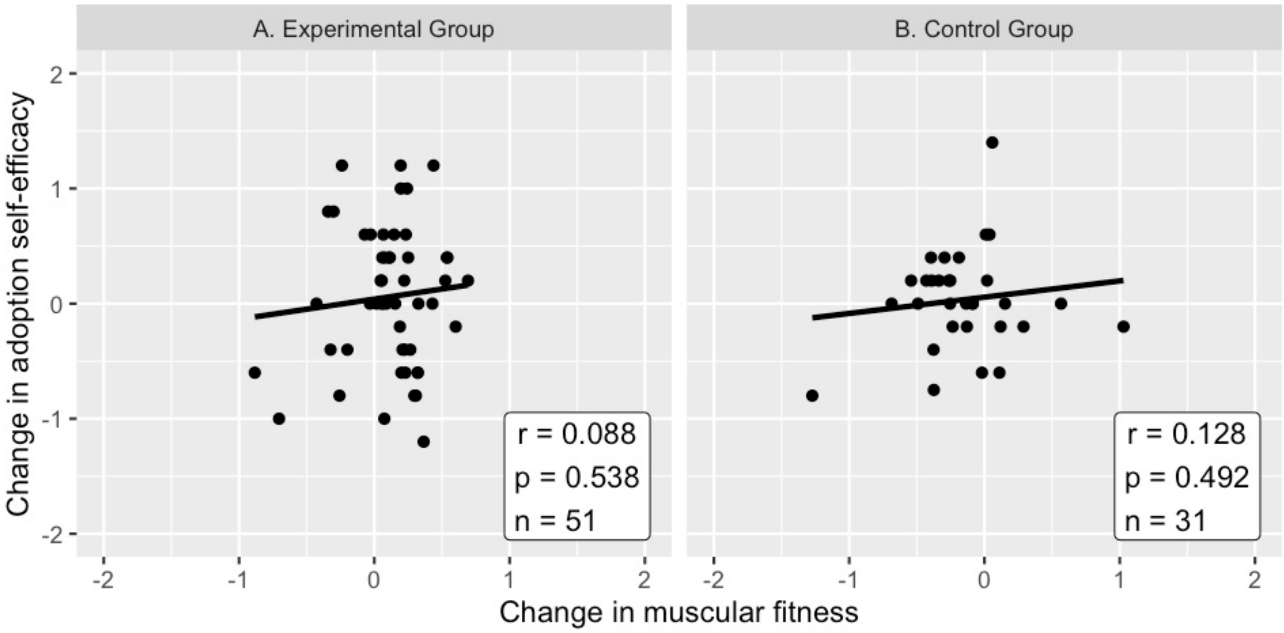

Supplementary Figure 2. A scatter plot for the association between changes in adoption self-efficacy and muscular fitness.

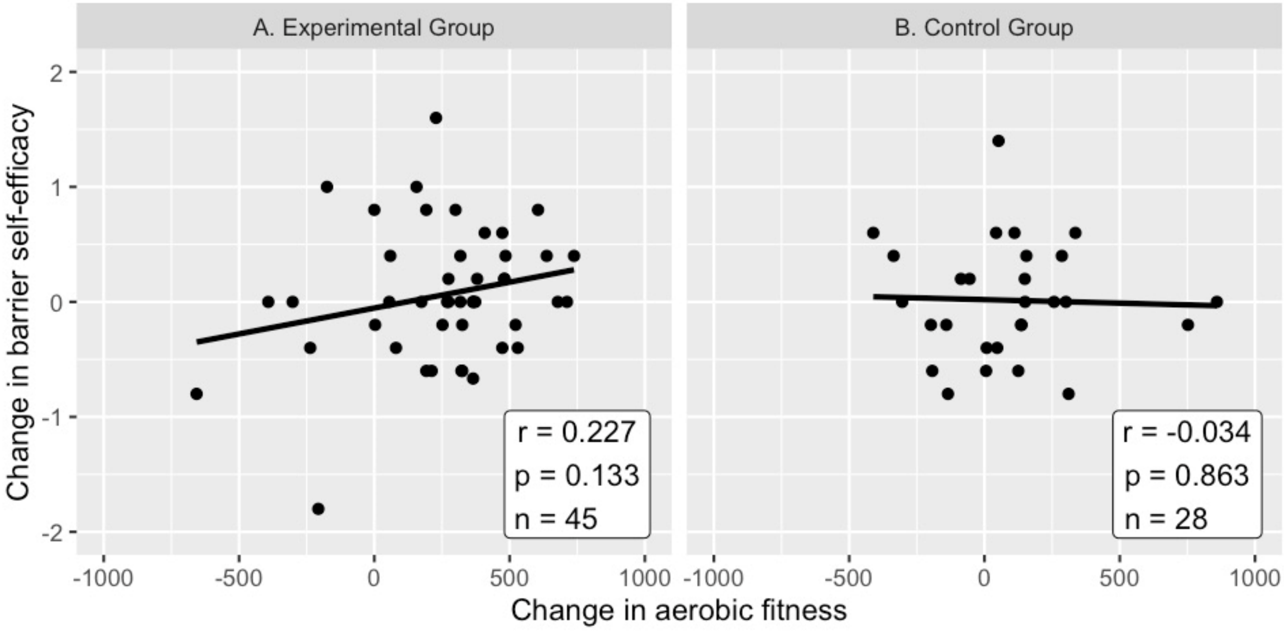

Supplementary Figure 3. A scatter plot for the association between changes in barrier self-efficacy and aerobic fitness.

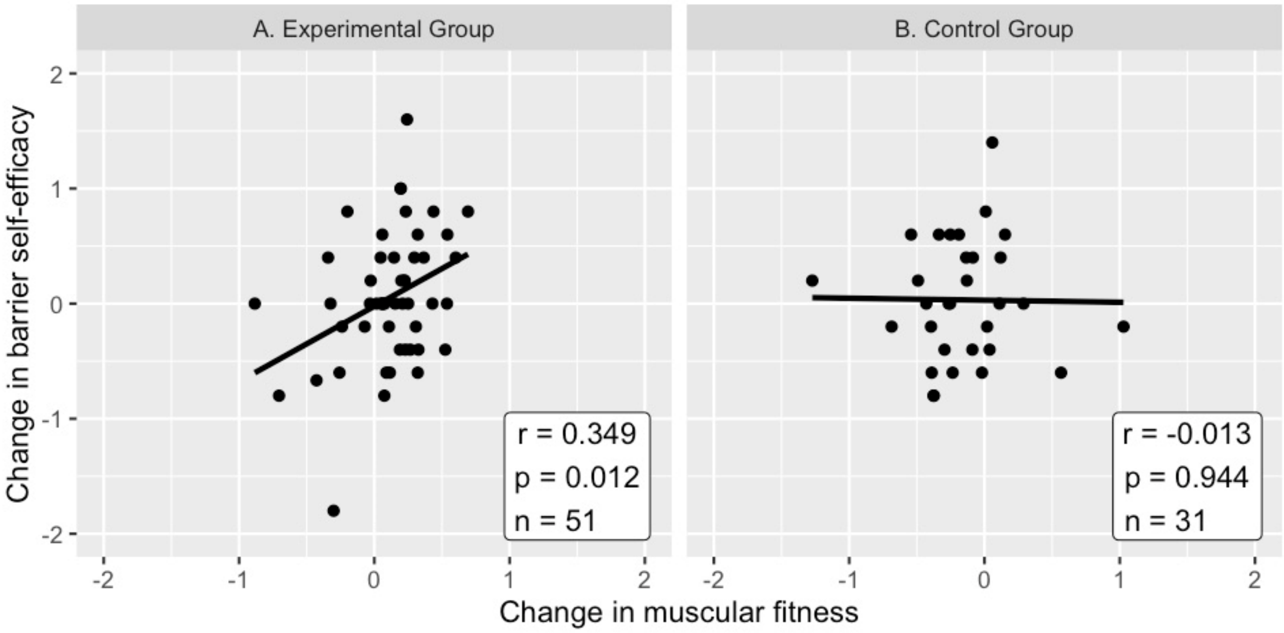

Supplementary Figure 4. A scatter plot for the association between changes in barrier self-efficacy and muscular fitness.
